# Impact of geographic accessibility on utilization of the annual health check-ups by income level in Japan: A multilevel analysis

**DOI:** 10.1371/journal.pone.0177091

**Published:** 2017-05-09

**Authors:** Misuzu Fujita, Yasunori Sato, Kengo Nagashima, Sho Takahashi, Akira Hata

**Affiliations:** 1 Chiba University Graduate School of Medicine, Department of Public Health, Chiba City, Chiba, Japan; 2 Chiba University Graduate School of Medicine, Department of Global Clinical Research, Chiba City, Chiba, Japan; 3 Chiba University Hospital, Clinical Research Center, Chiba City, Chiba, Japan; University of Saskatchewan, CANADA

## Abstract

Although both geographic accessibility and socioeconomic status have been indicated as being important factors for the utilization of health care services, their combined effect has not been evaluated. The aim of this study was to reveal whether an income-dependent difference in the impact of geographic accessibility on the utilization of government-led annual health check-ups exists. Existing data collected and provided by Chiba City Hall were employed and analyzed as a retrospective cohort study. The subjects were 166,966 beneficiaries of National Health Insurance in Chiba City, Japan, aged 40 to 74 years. Of all subjects, 54,748 (32.8%) had an annual health check-up in fiscal year 2012. As an optimal index of geographic accessibility has not been established, five measures were calculated: travel time to the nearest health care facility, density of health care facilities (number facilities within a 30-min walking distance from the district of residence), and three indices based on the two-step floating catchment area method. Three-level logistic regression modeling with random intercepts for household and district of residence was performed. Of the five measures, density of health care facilities was the most compatible according to Akaike’s information criterion. Both low density and low income were associated with decreased utilization of the health check-ups. Furthermore, a linear relationship was observed between the density of facilities and utilization of the health check-ups in all income groups and its slope was significantly steeper among subjects with an equivalent income of 0.00 yen than among those with equivalent income of 1.01–2.00 million yen (p = 0.028) or 2.01 million yen or more (p = 0.040). This result indicated that subjects with lower incomes were more susceptible to the effects of geographic accessibility than were those with higher incomes. Thus, better geographic accessibility could increase the health check-up utilization and also decrease the income-related disparity of utilization.

## Introduction

One of the highest priorities for health professionals and policy makers around the world is the provision of adequate health care resources [1, 2]. Nevertheless, inequalities of geographic accessibility among health care services, such as primary health care [3, 4], day care centers [5], tertiary care centers [6], and mammography screening [7–11], have been reported in a number of countries. To date, the impact of geographic accessibility on the utilization of health care services has not been evaluated thoroughly, and the results of previous studies are inconsistent. For example, some studies of mammography screening, the subject relatively frequently investigated, have found that low geographic accessibility to facilities was associated with low participation rates [8, 9, 12, 13], whereas others found no association or even an inverse association [10, 11, 14].

Socioeconomic status, such as income and education level, has been recognized as another factor in the utilization of health care services. Residents in deprived areas, determined by the low proportion of households owning houses, cars, and other goods, were less likely to receive treatment for colon or rectal cancer [15] or for breast cancer [16]. A study conducted in 21 member countries of the Organization for Economic Co-operation and Development found that low income was significantly negatively associated with visiting a medical specialist [17]. Recently, we also found decreased utilization of outpatient care among people with lower income in Japan [18].

The fact that both geographic accessibility and socioeconomic status are related to utilization of health care services prompted us to consider whether there is any interaction effect. The current study first confirmed the association between geographic accessibility and utilization of health check-ups in our target population and then analyzed the interaction between accessibility and income level. The subjects in this study were beneficiaries of National Health Insurance (NHI) in Chiba City and the response variable was utilization of the government-led nationwide annual health check-ups that started in April 2008 in accordance with the Act on Assurance of Medical Care for the Elderly by the Ministry of Health, Labour and Welfare. As municipalities in Japan, the insurers for NHI, are obliged to provide this program to all beneficiaries aged 40 to 74 years and to return the individual’s check-up data, complete data about the utilization by the beneficiaries of NHI in Chiba City are available. Information about income declared for tax purposes and about place of residence obtained from the basic residence registry was also provided by Chiba City Hall. The advantages of this study are its large sample size and accurate data that does not have the non-response bias that is unavoidable in questionnaire-based surveys.

## Materials and methods

### Subjects

In April 2008, the Japanese government launched an annual health check-up program targeting metabolic syndrome [19]. Under this program, insurers are obliged to provide a check-up to all beneficiaries aged 40 to 74 years. Japan has three major health care insurance systems [20]: a health insurance scheme for government and company employees; the NHI scheme administrated by municipalities for self-employed workers, farmers, retirees, and the unemployed; and a scheme for elderly adults aged 75 years or over. The subjects in this study were people in the second system in Chiba City. In fiscal year 2012, 167,115 beneficiaries of NHI in Chiba City were eligible for the check-ups. Of those, 149 (0.09%) were excluded because of missing data on residential address. In total, data on 166,966 people (78,627 men, 88,339 women) were analyzed.

### Ethics statement

Since this study was a retrospective observational study using existing data administrated by Chiba City Hall, consent was not obtained from each subject. All personal information (e.g., names and telephone numbers) was removed from the records, and all data were anonymized before being provided. This study was approved by the Research Ethics Committee of the Graduate School of Medicine, Chiba University. The study was conducted in accordance with the Declaration of Helsinki and the Ethical Guidelines for Medical and Health Research Involving Human Subjects.

### Response variable

The response variable of this study was utilization of the annual health check-ups during fiscal year 2012 (April 1, 2012, to March 31, 2013). Since the check-up is available only once a year for each subject, the outcome measure was binary (0 = non-participant; 1 = participant).

### Locations for the annual health check-up

In Japan, insurers have to set up annual health check-up sites for their beneficiaries. In fiscal year 2012, Chiba City set up 291 medical clinics and hospitals as facilities for the check-up. Beneficiaries of NHI in Chiba City were eligible to have the check-up once a year in any of the provided facilities from May 1, 2012 to February 28, 2013. The full address of these facilities could be obtained, which made it possible to construct point data for a geographic information system.

### Measurements of geographic accessibility

**Fig 1:**
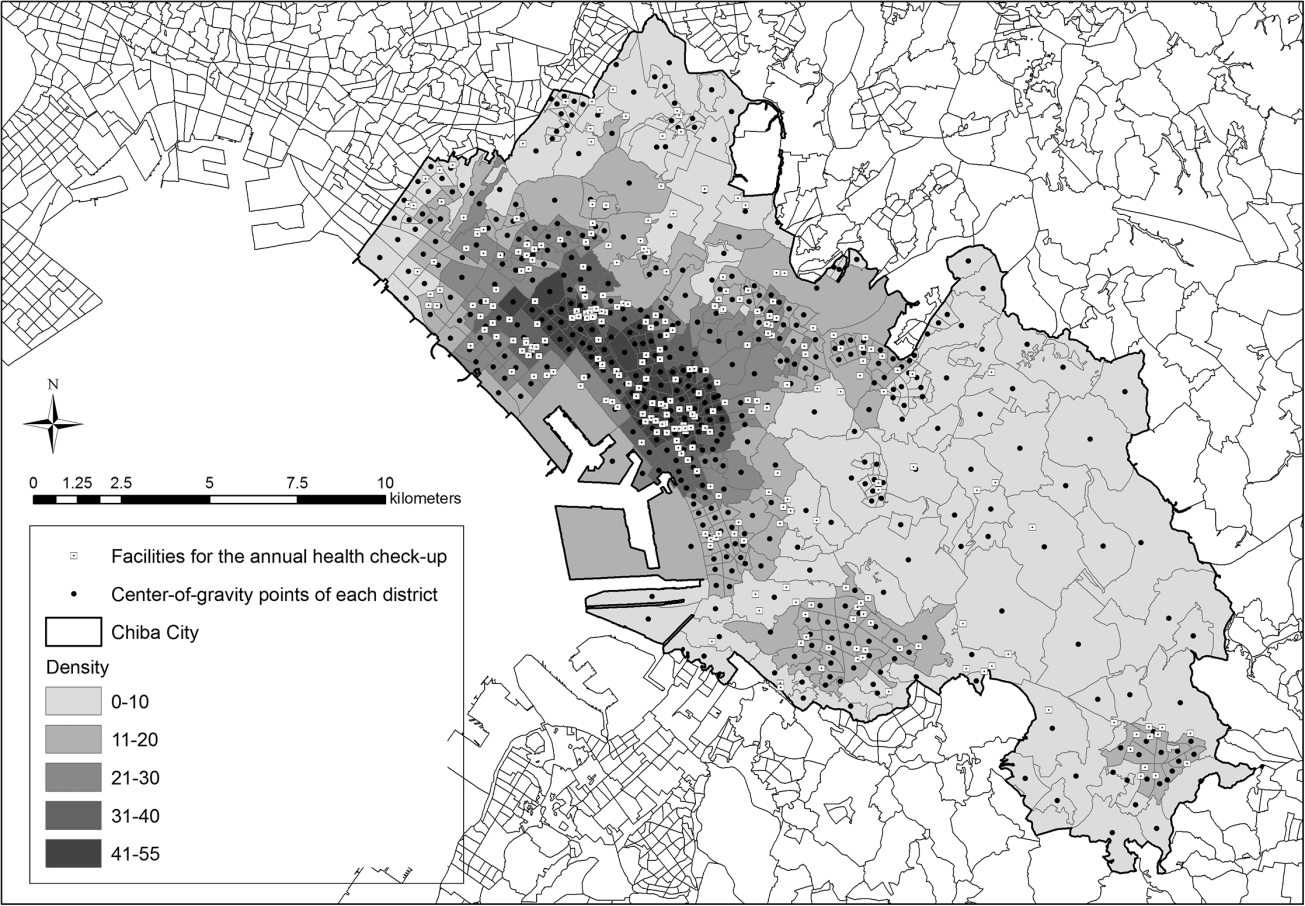
Locations of facilities for annual health check-ups and the center of gravity of each district.

We used resident registry data on town and district (the *chome* level) as provided by Chiba City Hall on April 2, 2012. The residential unit was at the district level, which has been used in national census data aggregation of small areas. In total, 494 districts were identified in Chiba City. We obtained the center of gravity of each district and assumed that all the beneficiaries in the district lived at this point. Fig 1 shows the center of gravity of each district and the location of each facility providing the check-up.

A total of five geographic accessibility measures—travel time to the nearest health care facility, density of health care facilities, an index based the two-step floating catchment area (2SFCA) method, an index based on the enhanced 2SFCA (E2SFCA) method with slow decay, and an index based on the E2SFCA method with quick decay—were calculated because no optimal index has yet been established [7, 21]. The Arc GIS version 10.1 geographic information system was used with the Arc GIS Network Analysis extension (ESRI Inc., Redland, CA) to calculate the measures. Road network data for Chiba Prefecture provided by ESRI Inc. were used for our purpose, and a walking speed of 4.8 km/h was assumed. The population for each district was obtained from 2010 Population Census of Japan data, which were included in the ArcGIS data-collection standard pack.

#### Travel time to the nearest health care facility

We calculated the walking time from the center of gravity of each district to the nearest facility provided for an annual health check-up.

#### Density of facilities for the annual health check-up

The areas of each district (the *chome* level) vary greatly, as shown in Fig 1: mean ± standard deviation was 0.54 ± 0.93 km^2^ and the minimum and maximum were 0.031 km^2^ and 6.9 km^2^, respectively. If we calculate the number of facilities for the check-up within this district as density, this measure greatly depend on the size of areas. Thus, we employed the number of facilities for the check-ups within a 30-min walking distance from the district of residence. Mean ± standard deviation of the areas of 30-min walking distance was 9.63 ± 1.49 km^2^ and minimum and maximum were 2.31 km^2^ and 13.3 km^2^, respectively.

#### Indices based on the 2SFCA and E2SFCA methods

We also applied the 2SFCA and E2SFCA methods, which are increasingly popular for estimating geographic accessibility in relation to the ratio of supply to demand [4, 7, 11, 22]. The 2SFCA method assumes that access does not diminish with distance within a catchment area. However, when a large catchment area is used, this assumption may not be appropriate. Thus, Luo and Qi (2009) introduced weights for different travel-time zones within a catchment area to account for distance decay. The method including these weights is referred to as the “E2DFCA” method. In this study, we calculated one index using the 2SFCA method and two indices using the E2SFCA method (E2SFCA with slow decay and E2SFCA with quick decay). The details have been given in previous studies [4, 22]. The procedure for calculating these indices is described briefly in the following paragraphs.

In the first step, the catchment area is defined as an area within a 30-min walking distance. Within each catchment area, three travel-time zones are used, with intervals of 0–10, 10–20, and 20–30 min (zones 1, 2, and 3, respectively). Different distance weights *W*_*r*_ were assigned to the three zones. The value of *W*_*r*_ is calculated from a Gaussian function in which access to the facility is assumed to diminish with increasing distance:

$$W_{r}=exp(\frac{{-(r-1)}^{2}}{\beta })$$

Here, *W*_*r*_ is the distance weight, *r* is the zone number (1, 2, or 3), and *β* is the impedance coefficient. In this study, we used *β* = 3.5 (*W*_*r*_ = 1.00, 0.75, and 0.32) for E2SFCA with slow decay and *β* = 1.5 (*W*_*r*_ = 1.00, 0.51, and 0.07) for E2SFCA with quick decay, as in a previous study [7]. For the 2SFCA calculation, *W*_*r*_ is not required.

With this setup, we found all population locations that are within a threshold travel-time zone from facility *j* (*D*_jr_) and computed the weighted facility-to-population ratio within the catchment area of the facility:

$$R_{j}=\frac{1}{\sum_{k\in \{d_{kj}\in D_{jr}\}}P_{k}W_{r}}$$

where *d*_*kj*_ is the travel time between facility *j* and district *k*, *D*_*jr*_ is the *r*th travel-time zone (*r* = 1, 2, or 3) within the catchment area of the facility, *P*_*k*_ is the population of district *k*, and *W*_*r*_ is the weight for each time zone.

In the second step, we found all facility locations *j* that are within the 30-min walking-time zone from all population locations *k*, and summed the facility-to-population ratios (*R*_*j*_ calculated in step 1):

$$A_{k}=\sum_{j\in \{d_{kj}\in E_{kr}\}}R_{j}W_{r}$$

Here, *A*_*k*_ is the index of geographic accessibility for the district at location *k*, *d*_*kj*_ is the travel time between *k* and facility *j*, *E*_*kr*_ is the *r*th travel time zone (*r* = 1, 2, or 3) within the catchment area from district location, *R*_*j*_ is the facility-to-population ratio at facility *j*, and *W*_*r*_ is the distance weight. A larger value of *A*_*k*_ indicates greater geographic accessibility.

### Income data

We used individual annual incomes for January 1 to December 31, 2012, as declared to Chiba City for tax purposes. The details of the income information has been described in a previous study [18]. Briefly, the number of people in a household was obtained by counting the number of people with the same household number, and the household income was calculated by summing the net incomes of the household members. The equivalent household income was calculated as household income divided by the square root of the number of household members [23]. We divided the subjects into four categories according to equivalent income (in millions of yen: 0.00, 0.01–1.00, 1.01–2.00, and 2.01 or more), and into three categories according to the number of family members (1, 2, and 3 or more).

### Statistical analysis

Summary statistics are expressed as frequencies and proportions for categorical data and as quartiles for continuous variables. For comparison between participants and non-participants in the check-ups, the Mann–Whitney U test was used for continuous variables and the chi-squared test was used for categorical variables. The association between geographic accessibility of the check-up facility and its utilization was analyzed using a multilevel mixed-effects logistic regression model (specifically, a three-level model with two random-intercept equations). Levels 1, 2, and 3 were at the individual (*n* = 166,966), family (*number of clusters* = 116,811), and residence district (*number of clusters* = 494) levels, respectively. Sex, age (continuous value), number of family members, and equivalent income were adjusted. Three measures (2SFCA, E2SFCA with slow decay, and E2SFCA with quick decay) had some extreme values, so values beyond the 99^th^ percentile were set to the value of the 99^th^ percentile. These thresholds were 5.87×10^−4^ for 2SFCA, 6.29×10^−4^ for E2SFCA with slow decay, and 8.97×10^−4^ for E2SFCA with quick decay. We determined the most suitable index for predicting utilization of the check-ups by using Akaike’s information criterion (AIC). For sensitivity analysis, a similar analysis was performed using the categorical variables of the indices for accessibility, grouped by quartile. Furthermore, we also examined a model including interaction between the index of accessibility and income on the utilization by using a multilevel mixed-effects logistic regression model. Results were considered significant when the two-tailed *p*-value was <0.05. All statistical analyses were performed using STATA software version 14 (Statacorp LP, College Station, TX).

## Results

A comparison of characteristics between participants and non-participants in the annual health check-ups is shown in Table 1. A total of 54,748 (32.8%) people had this check-up in fiscal year 2012. Participants have a tendency to be more elderly, more often female and have a higher income than non-participants. Compared with non-participants, the time required to reach the nearest facility from their district of residence was significantly shorter, their residential area had a significantly higher density of heath care facilities. The indices of 2SFCA, E2SFCA with slow decay, and E2SFCA with quick decay were also higher for participants.

**Table 1:** Comparison of characteristics for participants and non-participants in the annual health check-ups.

			Participants	Non-participants	p-value
Number^a^			54,748 (32.8)	112,218 (67.2)	
Age^b^	years		68 (63, 71)	64 (52, 69)	<0.001
Sex^a^					
		Men	22,260 (40.7)	56,367 (50.2)	<0.001
		Women	32,488 (59.3)	55,851 (49.8)	
Equivalent income^a^	million yen				
		0.00	6,647 (12.1)	20,892 (18.6)	<0.001
		0.01–1.00	15,941 (29.1)	34,759 (31.0)	
		1.01–2.00	20,171 (36.8)	33,570 (29.9)	
		2.01–	11,989 (21.9)	22,997 (20.5)	
Number of family members^a^					
		1	12,109 (22.1)	32,638 (29.1)	<0.001
		2	34,587 (63.2)	54,382 (48.5)	
		3 or more	8,052 (14.7)	25,198 (22.5)	
Shortest travel time^b^	minutes		6.1 (3.7, 9.9)	6.3 (3.8, 10.5)	<0.001
Density^b^	number of facilities		18 (9, 28)	17 (8, 26)	<0.001
2SFCA^b^	×10^−4^		3.0 (2.3, 3.9)	2.9 (2.2, 3.9)	<0.001
E2SFCA with slow decay^b^	×10^−4^		2.9 (2.2, 3.8)	2.8 (2.1, 3.8)	<0.001
E2SFCA with quick decay^b^	×10^−4^		3.0 (1.8, 4.2)	2.9 (1.7, 4.2)	0.002

^a^ Data shown as number (percent); chi-squared test was used.

^b^ Data shown as median (25^th^ percentile, 75^th^ percentile); Mann–Whitney U test was used.

The results of five models, one for each index for accessibility, using multilevel mixed-effects logistic regression analysis are shown in Table 2. In all models, people with higher income were more likely to utilize the check-ups. Shorter travel time to the nearest facility (odds ratio: 0.91; 95% confidence interval [CI]: 0.87 to 0.95; p < 0.001), higher density of health care facilities (odds ratio: 1.03; 95% CI: 1.01 to 1.04; p < 0.001), and larger E2SFCA with slow decay (odds ratio: 1.03; 95% CI: 1.00 to 1.06; p = 0.050) were significantly associated with higher utilization. In contrast, 2SFCA (odds ratio: 1.03; 95% CI: 1.00 to 1.06; p = 0.062) and E2SFCA with quick decay (odds ratio: 1.01; 95% CI: 0.99 to 1.04; p = 0.172) were not. According to AIC, the goodness of fit was best in the models including density of health care facilities or travel time to the nearest facility.

**Table 2:** Association of utilization of the annual health check-ups with equivalent income and indices for geographic accessibility using a multilevel mixed-effect logistic regression model.

			Index for accessibility														
			Travel time to the nearest facility			Density			2SFCA			E2SFCA with slow decay			E2SFCA with quick decay		
			OR	95% CI	p-value	OR	95% CI	p-value	OR	95% CI	p-value	OR	95% CI	p-value	OR	95% CI	p-value
**Fixed parameters**																	
**Individual factors**																	
	Sex																
		Men	1.00			1.00			1.00			1.00			1.00		
		Women	1.73	1.68–1.79	<0.001	1.73	1.68–1.79	<0.001	1.73	1.68–1.79	<0.001	1.73	1.68–1.79	<0.001	1.73	1.68–1.79	<0.001
	Age (year)		1.09	1.09–1.09	<0.001	1.09	1.09–1.09	<0.001	1.09	1.09–1.09	<0.001	1.09	1.09–1.09	<0.001	1.09	1.09–1.09	<0.001
**Household factors**																	
	Number of family members																
		1	1.00			1.00			1.00			1.00			1.00		
		2	1.50	1.43–1.58	<0.001	1.50	1.43–1.58	<0.001	1.50	1.43–1.58	<0.001	1.50	1.43–1.58	<0.001	1.50	1.43–1.58	<0.001
		3 or more	1.05	0.99–1.12	0.125	1.05	0.99–1.12	0.116	1.05	0.98–1.12	0.136	1.05	0.98–1.12	0.134	1.05	0.98–1.12	0.136
	Income (million yen)																
		0.00	1.00			1.00			1.00			1.00			1.00		
		0.01–1.00	1.37	1.29–1.46	<0.001	1.37	1.29–1.46	<0.001	1.37	1.29–1.46	<0.001	1.37	1.29–1.46	<0.001	1.37	1.29–1.46	<0.001
		1.01–2.00	1.96	1.84–2.09	<0.001	1.96	1.84–2.09	<0.001	1.96	1.84–2.09	<0.001	1.96	1.84–2.09	<0.001	1.96	1.84–2.09	<0.001
		2.01–	1.93	1.80–2.07	<0.001	1.93	1.80–2.07	<0.001	1.93	1.80–2.07	<0.001	1.93	1.80–2.07	<0.001	1.93	1.80–2.07	<0.001
**Contextual factors of residence**																	
	Indices for accessibility																
		Log travel time to the nearest facility	0.91	0.87–0.95	<0.001	-			-			-			-		
		Density^a^	-			1.03	1.01–1.04	<0.001	-			-			-		
		2SFCA^b^	-			-			1.03	1.00–1.06	0.062	-			-		
		E2SFCA with slow decay^b^	-			-			-			1.03	1.00–1.06	0.050	-		
		E2SFCA with quick decay^b^	-			-			-			-			1.01	0.99–1.04	0.172
			σ^2^	SE		σ^2^	SE		σ^2^	SE		σ^2^	SE		σ^2^	SE	
**Random parameters**																	
	Residence		0.360	0.018		0.356	0.019		0.364	0.019		0.364	0.019		0.367	0.019	
	Household		2.10	0.024		2.10	0.024		2.10	0.024		2.10	0.024		2.10	0.024	
AIC			189414			189414			189425			189425			189427		

Abbreviations. AIC: Akaike’s Information criterion; CI: confidence interval; OR: odds ratio, SE: standard error.

^a^ Density of health care facilities divided by 5 was entered in the model, so odds ratios are for increases of 5 in density.

^b^ 2SFCA, E2SFCA with slow decay, and E2SFCA with quick decay multiplied by 10^4^ were entered in the model, so odds ratios are for increases of 10^−4^ in these variables.

**Fig 2:**
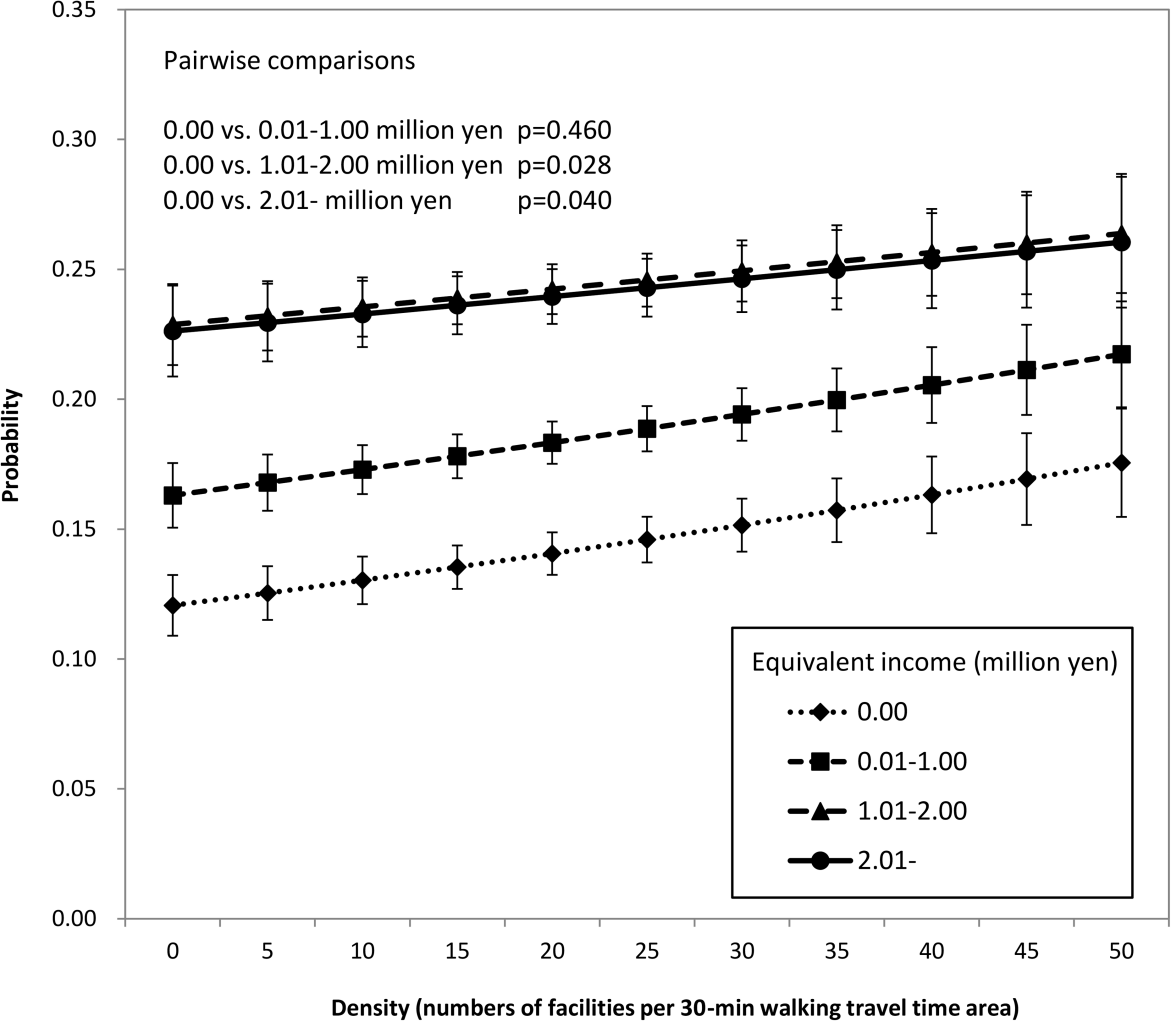
Probability of participation in the model including interaction between density of health care facilities and income.

We conducted sensitivity analyses in a similar way for categorical variables of accessibility indices, which were grouped by quartile, as shown in S1 Table. The model using density of health care facilities was found to have the smallest AIC, indicating the density (the number of facilities within a 30-min walk from the residential district) was the most suitable accessibility index for predicting participation in the check-ups in this study. Thus, we use this index hereafter. Fig 1 shows the districts of residence with different gray shades according to the density, the location of the facilities, and the center of gravity of each district. The results of the model including interaction between density of health care facilities and income are shown in S2 Table, and the probability predicted by this model is shown in Fig 2. A linear relationship was found between density of health care facilities and utilization of the check-ups in all income groups, indicating that better accessibility increased utilization of the check-ups in all income groups. In addition, the slope was significantly steeper for people with an equivalent income of 0 yen than for those with equivalent incomes of 1.01–2.00 million yen (p = 0.028) or 2.01 million yen or more (p = 0.040). This result indicates that people with lower income are more susceptible to the effects of geographic accessibility than those with higher income. In other words, income-dependent difference is large for a low density and small for a high density, as shown in Fig 2. When the density (in the model) was 0, the probability in the lowest income group was 0.121 and that in the highest group was 0.226, so the difference between them was 0.105. On the other hand, when the density was 50, the probabilities in the lowest and highest groups were 0.176 and 0.260, respectively, so the difference was 0.084. From these results, we suggest that better accessibility not only increased utilization of the check-up, but also decreased the difference of utilization between lower and higher income individuals.

In order to compare the goodness-of-fit of the models with and without accessibility, the Brier score and area under the receiver operating characteristic curve (AUC) were calculated. The Brier Score and AUC of the model with income and covariates (sex, age, and number of family members) were 0.0710 and 0.983 (95% confidence interval is 0.983–0.984, p<0.001), respectively, and those with additional variables of accessibility and interaction between income and accessibility were 0.0711 and 0.983 (95% confidence interval is 0.983–0.984, p<0.001). Thus, these indicators for the two models were very similar.

## Discussion

This study shows for the first time that both lower income and poor geographic accessibility decrease utilization of the government-led health check-ups in Japan. The significant interaction found between income and geographic accessibility indicates that people in a lower income group are more susceptible to the effects of geographic accessibility than those in a higher income group. Thus, better geographic accessibility could both improve health care service utilization and decrease the income-related disparity of utilization.

In Japan, the annual health check-ups, whose primary target is metabolic syndrome, was initiated in 2008 on a nationwide basis. Although the Japanese government set a specific target of 65% for participation in the check-ups for NHI by fiscal year 2012, the actual nationwide rate was 33.7%, similar to the 32.8% seen in our study population.

Various social factors play a significant role in the consultation behavior with no doubt. According to a theoretical model by the Institute of Medicine’s Committee on Monitoring Access to Personal Health Care Services, personal factors, such as income and education, financial factors, such as public support and insurance coverage, and structural factors, such as availability and transportation, all contribute to service utilization, which correlates with health status [24, 25]. Empirical observational studies have also shown that these three types of factor are associated with service utilization [9, 15, 18, 26] and health status [27, 28].

However, studies concerning measures to reduce socioeconomic status-related disparities in health care utilization are limited. Diderichsen et al. has proposed to dissect the approaches to improve an individual’s health behavior into individual oriented and structural oriented [29]. This categorization would be useful from an inequality standpoint, as in the former approach, the onus lies on each individual, while in the latter, the onus is on the political administrative system. It has been hypothesized that individual-oriented approaches (e.g., mass campaigns to change health behavior) increase socioeconomic status-related health inequality, since socially privileged people have more resources to effectuate the necessary efforts for changes in health behavior than do people who are socially disadvantaged [29]. On the other hand, a structural approach does not necessarily demand an active effort by individuals to change health behavior [29]. Thus, this approach could be an effective way to reduce disparity. For example, The State Children’s Health Insurance Program for 2,290 low-income children in New York City has been reported to reduce racial or ethnic disparities in medical care access [30]. In Japan, although the low-income group has a tendency not to use dental prostheses, the poorest people utilize the prostheses as frequently as those in the highest income group [31], taking into account the effect of public assistance (*seikatsu hogo*) that provides free dental care. In the current study, we have shown that better accessibility increases utilization overall and also decreases income-related differences. Thus, improving geographic accessibility, which is a structural approach, could contribute both to improved utilization and to decreased income-related disparities.

Although our result has shown a significant association between income and utilization and a significant interaction between income and geographic accessibility, these effect sizes seem to be small. Indeed, the Brier score and AUC of the models with and without geographic accessibility were quite similar. The large sample size in our study might have made a small relationship detectable. Thus, even if we intervene to increase geographic accessibility, a dramatic increase of utilization and decrease in income-related disparities might not occur.

There are several limitations in our study. First, the area of Ciba City is 271.8 km^2^, which is relatively small resulting in less variation of geographic accessibility. Nonetheless, a statistically significant (but small) association between accessibility and utilization was observed in the current study. Thus, an analysis with a larger area, including both urban and rural areas, could make the association even more important. Second, the assumption that all the beneficiaries lived at the center of gravity of their district (as nothing else can be done without a full address) could be a drawback, especially in large districts for which there could be a large possible deviation from the actual living point. Third, the current study examined the influence of geographic accessibility on the utilization of the annual health check-ups, which is only one among many health care services. In terms of other services, such as medical outpatient visits, dental visits, and cancer screening, the influence is unresolved. Fourth, we could not obtain other data related to socioeconomic status, such as occupation and education. For example, if many people with a specific occupation lived in regions where geographic accessibility was relatively low, then it might be occupation rather than the poor geographic accessibility that decreases utilization. Unavailable factors in the current study might confound the association between geographic accessibility and utilization.

## Conclusions

Better geographic accessibility increased the utilization of the annual health check-ups that is performed nationwide in Japan. In addition, individuals with lower incomes were more susceptible to the effects of geographic accessibility than were those with higher incomes. Better geographic accessibility might be an effective way to reduce income-related disparities in health care utilization.

## Supporting information

S1 Table Association between categorical value of accessibility of facilities and utilization of the annual health check-up: Multilevel mixed-effect logistic regression model. Abbreviations. AIC: Akaike’s Information criterion; CI: confidence interval; OR: odds ratio; SE: standard error.
(DOCX)

S2 Table Interaction between density and income with respect to utilization of the annual health check-up. Abbreviations. AIC: Akaike’s Information criterion; CI: confidence interval; OR: odds ratio; SE: standard error. ^†^ Density divided by 5 was entered in the model, so odds ratios are for an increase of density by 5.
(DOCX)
