# Supplementary material for: Impact of geographic accessibility on utilization of the annual health check-ups by income level in Japan: A multilevel analysis
Source: PLoS One. 2017 May 9;12(5):e0177091. doi: 10.1371/journal.pone.0177091 (PMC5423628; doi:10.1371/journal.pone.0177091)
Supplement: S1 Table — Abbreviations. AIC: Akaike’s Information criterion; CI: confidence interval; OR: odds ratio; SE: standard error. (DOCX) [file pone.0177091.s001.docx]

S1 Table. Association between categorical value of accessibility of facilities and utilization of the annual health check-up: Multilevel mixed-effect logistic regression model.

|  | Index for accessibility | | |  | | | |  | | |  | | |  | | |
| --- | --- | --- | --- | --- | --- | --- | --- | --- | --- | --- | --- | --- | --- | --- | --- | --- |
|  | Travel time to the nearest facility | | | Density | | | | 2SFCA | | | E2SFCA with slow decay | | | E2SFCA with quick decay | | |
|  | OR | 95% CI | p-value | OR | 95% CI | p-value | OR | | 95% CI | p-value | OR | 95% CI | p-value | OR | 95% CI | p-value |
| **Fixed parameters** |  |  |  |  |  |  |  | |  |  |  |  |  |  |  |  |
| **Individual factors** |  |  |  |  |  |  |  | |  |  |  |  |  |  |  |  |
| Sex |  |  |  |  |  |  |  | |  |  |  |  |  |  |  |  |
| Men | 1.00 |  |  | 1.00 |  |  | 1.00 | |  |  | 1.00 |  |  | 1.00 |  |  |
| Women | 1.73 | 1.68—1.79 | <0.001 | 1.73 | 1.68—1.79 | <0.001 | 1.73 | | 1.68—1.79 | <0.001 | 1.73 | 1.68—1.79 | <0.001 | 1.73 | 1.68—1.79 | <0.001 |
| Age (year) | 1.09 | 1.09—1.09 | <0.001 | 1.09 | 1.09—1.09 | <0.001 | 1.09 | | 1.09—1.09 | <0.001 | 1.09 | 1.09—1.09 | <0.001 | 1.09 | 1.09—1.09 | <0.001 |
| **Contextual factors of household** |  |  |  |  |  |  |  | |  |  |  |  |  |  |  |  |
| Number of family members |  |  |  |  |  |  |  | |  |  |  |  |  |  |  |  |
| 1 | 1.00 |  |  | 1.00 |  |  | 1.00 | |  |  | 1.00 |  |  | 1.00 |  |  |
| 2 | 1.50 | 1.43—1.58 | <0.001 | 1.51 | 1.43—1.58 | <0.001 | 1.50 | | 1.43—1.58 | <0.001 | 1.50 | 1.43—1.58 | <0.001 | 1.50 | 1.43—1.58 | <0.001 |
| 3 or more | 1.05 | 0.99—1.12 | 0.121 | 1.05 | 0.99—1.12 | 0.110 | 1.05 | | 0.98—1.12 | 0.134 | 1.05 | 0.99—1.12 | 0.131 | 1.05 | 0.98—1.12 | 0.135 |
| Income (million yen) |  |  |  |  |  |  |  | |  |  |  |  |  |  |  |  |
| 0 | 1.00 |  |  | 1.00 |  |  | 1.00 | |  |  | 1.00 |  |  | 1.00 |  |  |
| 0.01–1.00 | 1.37 | 1.29—1.46 | <0.001 | 1.37 | 1.29—1.46 | <0.001 | 1.37 | | 1.29—1.46 | <0.001 | 1.37 | 1.29—1.46 | <0.001 | 1.37 | 1.29—1.46 | <0.001 |
| 1.01–2.00 | 1.96 | 1.84—2.09 | <0.001 | 1.96 | 1.84—2.09 | <0.001 | 1.96 | | 1.84—2.09 | <0.001 | 1.96 | 1.84—2.09 | <0.001 | 1.96 | 1.84—2.09 | <0.001 |
| 2.01— | 1.93 | 1.80—2.07 | <0.001 | 1.93 | 1.80—2.07 | <0.001 | 1.93 | | 1.80—2.07 | <0.001 | 1.93 | 1.80—2.07 | <0.001 | 1.93 | 1.80—2.07 | <0.001 |
| **Contextual factors of residence** |  |  |  |  |  |  |  | |  |  |  |  |  |  |  |  |
| Index for accessibility |  |  |  |  |  |  |  | |  |  |  |  |  |  |  |  |
| Quartile 1 | 1.00 |  |  | 1.00 |  |  | 1.00 | |  |  | 1.00 |  |  | 1.00 |  |  |
| Quartile 2 | 1.01 | 0.90—1.13 | 0.882 | 1.26 | 1.12—1.41 | <0.001 | 1.25 | | 1.10—1.42 | <0.001 | 1.07 | 0.94—1.21 | 0.330 | 1.23 | 1.09—1.40 | 0.001 |
| Quartile 3 | 1.00 | 0.89—1.11 | 0.944 | 1.40 | 1.25—1.58 | <0.001 | 1.27 | | 1.13—1.43 | <0.001 | 1.22 | 1.08—1.38 | 0.002 | 1.24 | 1.10—1.40 | <0.001 |
| Quartile 4 | 0.80 | 0.71—0.90 | <0.001 | 1.37 | 1.23—1.52 | <0.001 | 1.16 | | 1.04—1.29 | 0.008 | 1.15 | 1.02—1.28 | 0.020 | 1.12 | 1.00—1.26 | 0.043 |
|  | σ^2^ | SE |  | σ^2^ | SE |  | σ^2^ | | SE |  | σ^2^ | SE |  | σ^2^ | SE |  |
| **Random parameters** |  |  |  |  |  |  |  | |  |  |  |  |  |  |  |  |
| Residence | 0.36 | 0.018 |  | 0.34 | 0.018 |  | 0.35 | | 0.019 |  | 0.36 | 0.019 |  | 0.36 | 0.018 |  |
| Household | 2.10 | 0.024 |  | 2.10 | 0.024 |  | 2.10 | | 0.024 |  | 2.10 | 0.024 |  | 2.10 | 0.024 |  |
| AIC^2^ | 189412 |  |  | 189393 |  |  | 189414 | |  |  | 189421 |  |  | 189417 |  |  |

Abbreviations. AIC: Akaike’s Information criterion; CI: confidence interval; OR: odds ratio; SE: standard error
