# Supplementary material for: Impact of geographic accessibility on utilization of the annual health check-ups by income level in Japan: A multilevel analysis
Source: PLoS One. 2017 May 9;12(5):e0177091. doi: 10.1371/journal.pone.0177091 (PMC5423628; doi:10.1371/journal.pone.0177091)
Supplement: S2 Table — Abbreviations. AIC: Akaike’s Information criterion; CI: confidence interval; OR: odds ratio; SE: standard error. † Density divided by 5 was entered in the model, so odds ratios are for an increase of density by 5. (DOCX) [file pone.0177091.s002.docx]

S2 Table. Interaction between density and income with respect to utilization of the annual health check-up.

|  | OR | 95% CI | p-value |
| --- | --- | --- | --- |
| **Fixed parameters** |  |  |  |
| **Individual factors** |  |  |  |
| Sex |  |  |  |
| Men | 1.00 |  |  |
| Women | 1.73 | 1.68—1.79 | <0.001 |
| Age (year) | 1.09 | 1.09—1.09 | <0.001 |
| **Contextual factors of household** |  |  |  |
| Number of family members |  |  |  |
| 1 | 1.00 |  |  |
| 2 | 1.51 | 1.43—1.58 | <0.001 |
| 3 or more | 1.05 | 0.99—1.12 | 0.114 |
| Income (million yen) |  |  |  |
| 0 | 1.00 |  |  |
| 0.01–1.00 | 1.42 | 1.27—1.58 | <0.001 |
| 1.01–2.00 | 2.16 | 1.94—2.41 | <0.001 |
| 2.01— | 2.13 | 1.89—2.40 | <0.001 |
| **Contextual factors of residence** |  |  |  |
| Density^†^ | 1.04 | 1.02—1.07 | <0.001 |
| **Individual and residence interaction** |  |  |  |
| Interaction between density and income |  |  |  |
| Density×income 0.01—1.00 | 0.99 | 0.97—1.01 | 0.460 |
| Density×income 1.01—2.00 | 0.98 | 0.95—0.99 | 0.028 |
| Density×income 2.01— | 0.97 | 0.95—0.99 | 0.040 |
|  | σ^2^ | SE |  |
| **Random parameters** |  |  |  |
| Residence | 0.35 | 0.019 |  |
| Household | 2.10 | 0.024 |  |
| AIC | 189412 |  |  |

Abbreviations. AIC: Akaike’s Information criterion; CI: confidence interval; OR: odds ratio; SE: standard error

^†^ Density divided by 5 was entered in the model, so odds ratios are for an increase of density by 5.
